# How Gig Worker Responds to Negative Customer Treatment: The Effects of Work Meaningfulness and Traits of Psychological Resilience

**DOI:** 10.3389/fpsyg.2021.783372

**Published:** 2021-12-08

**Authors:** He Xiongtao, Lu Wenzhu, Luo Haibin, Liu Shanshi

**Affiliations:** ^1^School of Business Administration, South China University of Technology, Guangzhou, China; ^2^Guangdong Women’s Polytechnic College, Guangzhou, China

**Keywords:** gig economy workers, negative customer treatment, positive customer treatment, work meaningfulness, psychological resilience

## Abstract

The negative interpersonal interaction between customers and platform gig workers has become a problem for platform owners and government. This study investigates the role of negative customer treatment in the context of gig work and its impact on gig workers’ sabotage behavior. A questionnaire survey approach was used in the study, collected three-wave survey data from 258 Chinese gig workers including food-deliver platform workers and app-based ride-hailing drivers. Both effects of the mediation and moderation were tested, all of which find support, using hierarchical multiple regression by SPSS22.0. Results indicate that negative customer treatment can also predict gig workers’ service sabotage through work meaningfulness. Furthermore, positive customer treatment acted as an effective safeguard against the effects of negative customer treatment on employee service sabotage. Trait psychological resilience can also mitigate the effects of a low level of work meaningfulness. The manuscript’s focus provides an interesting angle to the previous research, especially the inclusion of work meaningfulness and trait resilience, on negative customer treatment in the context of gig work. This study contributes to further broaden the perspective of conservation of resource (COR) theory for individual intrinsic motivation analysis. Practical implications for platform management and government governance have also been discussed in this manuscript.

## Introduction

Gig workers are increasingly common, as the sharing economy provides plenty of employment opportunities. The explosive growth of online labor platforms has attracted many gig workers in the service industry (Lo Presti et al., 2018). The rapid growth of the sharing economy has caused us to consider the phenomenon of gig workers being exposed to negative customer treatment during service interactions—i.e., customers treating employees in disrespectful, demeaning, unreasonable or aggressive ways (Zhan et al., 2016). For gig workers, they experience particularly high levels of negative customer treatment because they more frequently interact with customers and provide services for them. The negative interpersonal interactions between dependent contract workers and customers hampers the development of the platform; as a result, numerous scholars have allocated more attention to exploring this phenomenon.

Recent studies have also called for more research to explore gig workers’ organizational behaviors (Guillaume et al., 2019). Existing literature has already found that negative customer treatment had important difference on full-time employees. However, an empirical examination on the effects of negative customer treatment is largely missing from the perspective of gig workers and the underlying mechanisms of positive psychology. Is it possible that negative customer treatment may also lead to negative outcomes for gig workers? Furthermore, should gig workers who experienced negative customer treatment also choose to engage in sabotage against customers, what mechanism would explain this phenomenon? How to minimize the damage on the customer experience? Would positive psychology play the role? Our research intends to respond to these practical needs.

The first objective of this study is to identify the mechanisms of interaction between gig workers’ sabotage behavior and negative customer treatment. It is significant to advance the understanding of the link between negative customer treatment and its impact on gig workers’ sabotage behavior in the context of gig work. Previous research has demonstrated that when full-time workers experience negative customer treatment, they may engage in service sabotage that extremely damaged to customer experience and organizations’ reputations (Rupp et al., 2008; Wang et al., 2013; Amarnani et al., 2019). In contrast with the full-time workers, gig workers take charge of their own career development and poor employment relationships with the various available platforms (Sammarra et al., 2013). It is difficult for the platform to directly manage gig workers’ behaviors. Thus, gig workers might more freely express their behaviors when exposed to negative customer treatment.

For some gig workers, they engage in career self-management and are prone to act as owners and agents of their own careers (Sammarra et al., 2013). Customer satisfaction is closely related to their own earnings and influences their reputation on the platform (McKeown and Pichault, 2020). Even when confronted with negative customer treatment, they may not be likely to engage in service sabotage like full-time workers. So research progress will require further understanding of how gig workers respond to negative customer treatment compared to full-time workers.

The second objective of this study is to test the conservation of resource (COR) theory (Brotheridge and Grandey, 2002) in perspective of positive psychology. Based on the COR theory, individuals are always inclined to acquire, maintain, protect, and cultivate their resources. Workers who cannot feel the meaning of the work will consider it a kind of resource loss (Hobfoll, 2001). Gig workers put their finite resources into interaction with customers to obtain customer recognition and praise of their service (Brotheridge and Grandey, 2002). The improvement of work meaningfulness means that individuals can obtain resources through interaction with customer service. In order to further obtain resources, individuals will give full efforts to their potential to serve customers and establish good interpersonal relationships with customers. Gig workers who have perceived higher work meaningfulness have no reason to behavior service sabotage.

Few researchers have explored its effect from the positive psychology depletion mechanism. The negative interpersonal interactions between gig workers and customers hampers the development of the platform. As a result, numerous scholars have allocated more attention to exploring this phenomenon. For example, Wang et al. (2013) revealed that customer treatment can have a negative effect on one’s mood, which is based on cognitive theories of rumination. However, Yue et al. (2017) suggested that negative customer treatment predicted coworkers helping from the perspective of negative state relief theory. For gig workers, work is not only a means of earning a living but also a way to gain meaningfulness. When individuals experience negative customer treatment, they may question the significance and the meaning attached to the work (Loi et al., 2018). Therefore, it is imperative to explore the positive psychological mechanisms of work meaningfulness in terms of negative customer treatment and service sabotage in the context of the gig economy.

Several potential theoretical contributions are included in this study. First, the present study addresses gaps to verify the effective mechanisms of positive psychology that prior research not concerned. Existing research mainly focused on the dark side of the interaction between the customer and employee, i.e., how negative customer treatment influences service outcome and individual well-being (Wang et al., 2013; Zhan et al., 2016; Amarnani et al., 2019; Arvan et al., 2020). But during the service process, there also exist positive aspects of the employee-customer’s interaction. Positive customer treatment, such as smiling and appraising, makes employees aware of being respected. This pleasant experience may prohibit the effect of negative customer treatment. Therefore, prior research focused only on negative customer treatment, which limited scholars’ understanding of customer interaction completely. In our research, we suggest that both negative and positive customer treatment toward service workers can influence employees’ service sabotage.

Second, the topic of negative customer treatment in the context of gig work is intriguing, and the research’s focus provides an interesting angle to the previous research. It is critical for organizations to enhance the customers’ experience. Considering the large damage of negative interpersonal interaction, it is necessary to explore the boundary condition in terms of negative customer treatment and individuals’ service sabotage. This study focuses on investigating how negative customer treatment influences gig workers’ responses. We argue that encountering misbehaved customers decreased gig workers’ work meaningfulness, which in turn, motivated them to retaliate against customers. Furthermore, we suggest that positive customer treatment can mitigate the increased service sabotage related to negative customer treatment. We also predicted that individual psychological resilience traits can prevent individuals from suffering low levels of work meaningfulness resulting from negative customer treatment.

**FIGURE 1:**
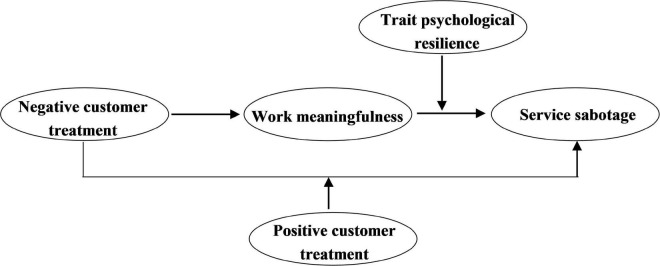
The proposed model.

Third, the present study contributes to the negative customer treatment literature in several ways. Although some studies have found the negative impact of customer negative treatment on employees (Lin and Lai, 2020; Lee and Madera, 2021). The study broadened the research context by investigating the coping strategies of gig workers in the gig economy. Furthermore, from the perspective of the positive psychological mechanism, we revealed the mediating mechanism of work meaningfulness in terms of the relationship between negative customer treatment and service sabotage for gig workers. Prior studies have focused on the negative effects of this mechanism (Cheng et al., 2020; Lee et al., 2020). Our work further reveals the “black box” between negative customer treatment and employee behavior. Finally, we argue that positive customer treatment and trait psychological resilience can mitigate the effect of negative customer treatment from the conservation theory of resources. Figure 1 depicts our conceptual model.

Therefore,on the basis of the above explanation, the academic research questions (ARQ) are developed below.

ARQ1: How negative customer treatment influences the gig workers’ service sabotage?

ARQ2: How does positive customer treatment intervene between negative customer treatment and the service sabotage?

ARQ3: How does trait psychological resilience intervene between work meaningfulness and the service sabotage?

## Theoretical Background and Hypotheses

### Negative Customer Treatment and Service Sabotage

Negative customer treatment is defined as customers treating employees in an unreasonable, belittling, aggressive or disrespectful way (Skarlicki et al., 2008), covering multiple kinds of low-quality interpersonal communications between employees and customers (Wang et al., 2011). The negative treatment including verbal abuse, unreasonable demands and rude behavior that customers inflict on service workers (Amarnani et al., 2019). Previous studies have proposed different terms about the negative interactions between customers and service employees, such as customer bullying, customer rudeness and customer misbehavior. It is necessary to define the difference among these terms before the following analysis. Customer bullying refers to the behavior that customers engage in violation of consumption habits and social norms for profit as well as the low-quality interpersonal treatment (Zhan et al., 2015). Evolving from workplace incivility, customer rudeness refers to low-intensity deviant behavior with vague intent to harm the target, and in violation of norms of mutual respect in the workplace (Sliter et al., 2012). Customer misbehavior refers to the problem that the customer’s behavior intentionally violates the generally accepted code of conduct regarding how to treat employees in the process of service, emphasizing the problem of intention and normative deviation (Daunt and Harris, 2012). It can be seen that the above concepts overlap with each other but each has its own focus, while negative customer treatment is an umbrella construct that covers the various forms of low-quality interpersonal interaction that employees receive from customers (Wang et al., 2011).

Service sabotage is defined as any open or secret deviant behavior that service employees intentionally take to destroy the quality of customer service and harm the interests of customers (Harris and Ogbonna, 2006; Wang et al., 2011; Groth and Grandey, 2012; Chi et al., 2018). The behavior is a type of deviant behavior that includes intentionally changing the speed of service, expressing dissatisfaction, frustration or hostility toward a customer, retaliating against rude customers and intentionally overcharging customers for services provided to them (Harris and Ogbonna, 2006). The negative outcome of service sabotage has been confirmed, such as reducing customer satisfaction and loyalty as well as the long-term profitability of the organization (Harris and Ogbonna, 2006; Skarlicki et al., 2008; Wang et al., 2011; Groth and Grandey, 2012).

Although several studies have shown the positive relationship between negative customer treatment and service sabotage in traditional service industries, such as the hairdressing industry (Chi et al., 2013), call center industry (Skarlicki et al., 2008), catering industry (Tao et al., 2019), and hotel industry (Cheng et al., 2020). Less is known about the relationship in the context of the gig economy. To address this research gap, this study focuses on the relationship between negative customer treatment and service sabotage. Due to the service nature of gig workers, it is inevitable that they will be subject to mistreatment by customers in the process of providing services. Because no matter what countermeasures gig workers take, irrational, and improper customers will always exist (Harris and Ogbonna, 2006). Therefore, this study argues that, for workers in the gig economy, negative customer treatment will also lead to service sabotage.

First, the traditional mode of employment services usually emphasizes that “the customer is god,” and “the customer is always right” (Lee et al., 2020; Park and Kim, 2020). Even if the employees are mistreated by customers, they are expected to suppress negative emotions and express a positive attitude and behavior to obey the rules of the organization (Grandey et al., 2004). Otherwise, employees may be subject to disciplinary actions for harsh responses to uncivilized customers (Groth and Grandey, 2012). Therefore, under the traditional employment model, the mandatory regulation and control of service organizations on employees might reduce the direct service sabotage of employees to customers’ mistreatment. However, gig workers are not considered as the real employees of platform firms (Kuhn and Maleki, 2017). Therefore, while facing negative customer treatment, gig workers are more likely to engage in direct service sabotage behavior because of the lack of formal organizational norms and rules. Secondly, in the service industry under the traditional employment model, it is generally believed that customers have the right to make demands which motivate service organizations to satisfy them. Consequently the motivation leads to the fact that customers usually enjoy greater rights than employees (Hochschild, 2012), reflecting the social status gap between customers and service providers to a certain extent. This gap may lead employees to view negative customer treatment as acceptable to a degree that does not trigger retaliation. However, for gig workers, the individual status changes from being employed to self-employed, individuals provide services through the platform, and the relationship between gig workers and customers is limited to a simple buying and selling relationship. This new employment model helps to reduce the inequality between gig workers and customers. As a result, gig workers are more sensitive to negative customer treatment, perceived as unfair interpersonal treatment, and more likely to sabotage their services. To summarize, this study proposes the following hypothesis:

H1: Negative customer treatment is positively related to service sabotage for gig workers.

### Mediating Role of Work Meaningfulness

Work meaningfulness refers to individuals considering the importance and intrinsic value of their work based on their subjective experience (Rosso et al., 2010). Work has increasingly become a key area for individuals to obtain the meaning of life (Steger et al., 2012) and a major aspect of employees’ intrinsic motivation (Humphrey et al., 2007). Although, some scholars have suggested that people working in the hospitality industry work to make a living (Huang et al., 2004; Jung and Yoon, 2016), it cannot explain why some workers still claimed to continue working even if they won lottery or inherited a sum of money large enough to support themselves in the future (Harpaz and Fu, 2002). Extensive discussions around the motivation of gig workers have suggested that besides obvious financial motives, gig workers are more willing to enhance and maintain their sense of self (Abubakar and Shneikat, 2017).

In the process of providing services, the interpersonal interaction between gig workers and customers is the main factor that affects the meaning of work (Bailey et al., 2017). When gig workers face negative customer treatment, the meaning of their work might be doubted (Loi et al., 2018), preventing them from getting meaning out of their work. Meanwhile, the negative treatment of customers devalues the personal value of gig workers (Park and Kim, 2020), threatening their self-concept and leading to the decrease of their work significance (Wang et al., 2011). Based on the COR theory perspective, gig workers put their finite resources into interaction with customers to obtain customer recognition and praise of their service (Brotheridge and Grandey, 2002). Customers’ mistreatment behavior will make gig workers unable to get the benefit from the resources they invested; they will find their own work did not bring value to others and themselves, thus resulting in a decline in perceived work meaningfulness.

Given that work meaningfulness is the most critical psychological factor that connects society, work characteristics, and work outcomes (Humphrey et al., 2007). This study indicates that gig workers who have experienced more negative customer treatment will perceive lower work meaningfulness and further lead to service sabotage. First, according to COR theory, individuals are always inclined to acquire, maintain, protect and cultivate their resources (Hobfoll, 2001). Workers who cannot feel the meaning of the work will consider it a kind of resource loss (Hobfoll, 2001). To recover the lost resources, service sabotage is adopted by gig workers (Skarlicki et al., 2008; Wang et al., 2011). Second, the improvement of work meaningfulness means that individuals can obtain resources through interaction with customer service. In order to further obtain resources, individuals will give full play to their potential to serve customers and establish good interpersonal relationships with customers. Gig workers who have perceived higher work meaningfulness have no reason for behavior service sabotage. That is, work meaningfulness has a negative effect on service sabotage. It can be concluded from the above analysis that work meaningfulness might be the positive psychological mechanism linking negative customer treatment and service sabotage. To sum up, this study puts forward the following hypothesis:

H2: Work meaningfulness mediates the effect of negative customer treatment on gig workers’ service sabotage.

### Moderating Role of Positive Customer Treatment

In contrast to negative customer treatment, positive customer treatment means that customers treat employees with gratitude and respect (Zhan et al., 2015). The positive treatment of customers is pleasant and rewarding for the service staff, which can trigger positive emotional experiences and reduce the emotional loss of the employees (Brotheridge and Grandey, 2002). Existing literature has focused on exploring the negative interaction between customer and service employee. However, a lack of empirical studies to examine the positive side of interaction (for example, the positive treatment of customers) between customers and service workers might not fully explain the mechanism in terms of negative customer treatment and service sabotage.

There are some reasons for the lack of studies exploring the positive interaction between customers and workers. First, the notion of affect symmetry argues that positive traits and emotions predict positive outcomes, while negative traits and emotions predict negative outcomes (Bono et al., 2013). Second, people have an innate propensity to remember negative things, so it is natural to consider the role of negative customer treatment in exploring the antecedents of service sabotage. But Thoresen et al. (2003) conducted a meta-analysis that challenged the proposal of affect symmetry, suggesting that a positive attitude can have the same effect on negative and positive outcomes. At the same time, in our daily lives, we can also notice that customers also treat servicers in a polite way. Therefore, the role of positive treatment of customers should not be ignored.

This study argues that positive customer treatment can effectively weaken the positive relationship between negative customer treatment and service sabotage. First, based on COR theory, the increase of resources can reduce the negative impact caused by resource loss (Hobfoll, 2001). Positive customer treatment is a positive experience for gig workers, which can increase individual core resources and counteract the pressure generated by negative customer treatment, thus reducing service sabotage. Second, for gig workers, positive customer treatment means beneficial experience to construct resources indirectly by satisfying the basic needs of gig workers (Zhan et al., 2015), including the sense of belonging and autonomy. These resources can restore the resource loss caused by customers’ mistreatment (Lilius, 2012), further effectively reducing service sabotage behavior. Therefore, this study puts forward the following hypothesis:

H3: Positive customer treatment negatively moderates the relationship between negative customer treatment and service sabotage, such that the negative relationship is weaker when gig workers perceive more positive customer treatment.

### Moderating Role of Psychological Resilience

Psychological resilience refers to the ability of individuals to effectively cope with adversity and pressure in the face of difficulties and setbacks to achieve sound physical and psychological development (Luthans and Youssef, 2007). Research has shown that psychological resilience not only plays a role in the major events experienced by individuals, but also affects the ability of individuals to cope with daily trifles (Hobfoll, 2001). Studies about negative customer treatment show that individuals adopt various strategies to deal with negative customer treatment (Li et al., 2019), and not all employees will show negative work consequences when they encounter negative customer treatment (Tao et al., 2019). Therefore, this study proposes that the psychological resilience of individuals may effectively alleviate service sabotage caused by the decrease of work meaningfulness.

First, according to COR theory, compared with employees with lower resilience, employees with higher psychological resilience regard their resilience as a kind of psychological resource. Individuals with more psychological resources can better face the pressure brought about by the reduction of work meaningfulness and then reduce the service sabotage behavior (Hobfoll, 2001). Second, according to the definition of psychological resilience, psychological resilience can help individuals better withstanding pressure and recover from adversity. Gig workers with higher psychological resilience are more likely to actively seek help and take effective ways to solve problems when their work meaningfulness is reduced, thus generating optimistic attitudes and low levels of pressure (Cooper et al., 2014), reducing service sabotage caused finally. To sum up, this study puts forward the following hypothesis:

H4: Psychological resilience positively moderates the relationship between work meaningfulness and service sabotage.

## Research Methodology

### Research Approach

In the empirical research part of this article, we used the questionnaire survey approach. Questionnaire surveys are often used to obtain data when conducting empirical research. The more rigorous the process of measuring questionnaire design, the more explanatory the research results. Therefore, rigorous questionnaire design and scientific investigation process will help improve the reliability and validity of the research and obtain credible research results and conclusions. So, we have carefully designed the questionnaire.

### Instrument Development

In this manuscript, we used negative customer treatment as independent variable, service sabotages as dependent variable, work meaningfulness as the mediator, positive customer treatment, and psychological resilience as the moderators. The first section of the instrument describes the purpose of the study and contained instructions for replying, as well as anonymity and privacy statements. The second section of the instrument consists of the respondents’ personal information (gender, experience, marital status, age, and education). The third part describes the items of the selected variables. Before the formal survey, we conducted a small sample pre-test, distributed 60 questionnaires, and recovered 48 valid questionnaires. Cronbach’s alpha and principal component analysis is used to verify the reliability and effectiveness of the scale. The analysis of 48 valid samples shows that the reliability values of the four scales involved are all greater than 0.7, and the factor loading values are all greater than 0.5. It shows that the scale has good reliability and validity.

### Variable Measures

Drawing on the experience and practices of most scholars, the measurement of relevant variables in this study adopts mature scales published. All scale items were in the form of a statement by a Likert 5-point scoring system ranging from 1 (strongly disagree) to 5 (strongly agree). In order to ensure the validity of the scales in the Chinese context, all English scales in this research were translated using Brislin (1970) back-translation procedures.

Negative customer treatment was measured using the scale developed by Wang et al. (2011) and consisted of 12 items (for example, “customers yell at me”). Subsequent studies have also confirmed the reliability and validity of the scale in the Chinese context (Zhan et al., 2015). In this study, the reliability was good (Cronbach’s α = 0.87).

Positive customer treatment was measured using the interpersonal fairness developed by Colquitt (2001) and the daily positive events scale of Oishi et al. (2007). A sample item read “received additional rewards from customers.” Again, the reliability was good (Cronbach’s α = 0.82).

Work meaningfulness was measured by the scale developed by Steger et al. (2012) and consisted of 10 items. A sample item read “I find that work will make me feel satisfied.” The study’s reliability was good (Cronbach’s α = 0.88).

Psychological resilience was measured using the resilience dimension of the psychological capital scale developed by Luthans and Youssef (2007). This measurement instrument consists of six items. A sample item read “I usually deal with the stress of work calmly.” In this study, the reliability was adequate (Cronbach’s α = 0.80).

Service sabotages were measured using the scale developed by Chi et al. (2013), and they consisted of six items. A sample item read “I behave very negatively to customers.” Subsequent studies have also confirmed the reliability and validity of the scale in the Chinese context (Li et al., 2019; Cheng et al., 2020). Reliability for this scale was good (Cronbach’s α = 0.90).

Following previous research (Wang et al., 2011; Zhan et al., 2015), this study will control the basic demographic variables of individuals, including gender, age, marital status, educational background and working years.

### Sample and Procedures

We obtained data from food-deliver platform workers and app-based ride-hailing drivers. The selection of the above-mentioned research objects is mainly based on the following considerations. The online car-hailing and food delivery industries have become the most representative industry in the gig economy. Platform owners and government officers notice that many problems arise from the negative interpersonal interaction between customers and platform gig workers. For example, online car-hailing drivers often make detours, dump passengers and abuse passengers; the food delivery workers deliberately delay the delivery time, knock over food and even harass customers and other negative news. Those problems do harm to customer experience and the society stability. The research on the above objects has urgent practical significance.

To alleviate the potential common method variance concern, our study collected the data at different points in time (1 month apart). At time 1, we measured the demographic information and the variable of customer mistreatment; a total of 423 questionnaires were collected. At time 2, we measured the variables of customer positive treatment and psychology resilience, and a total of 384 questionnaires were collected. At time 3, we measured the variables of work measured and service sabotage. A total of 355 questionnaires were collected. All data was collected by online surveys.

After the data was collected, we deleted the samples, such as a short answering time. Finally, 258 valid questionnaires remained (61% response rate). The descriptive statistics of the sample show that the participants were mainly male (63.7%), 68.6% of the participants were 30 years old and below, 68.2% of the participants had worked less than 3 years, 33.0% of the participants were unmarried, and 79.8% of the participants were junior college students.

## Results

### Reliability and Validity of the Construct

Although this study adopted three-time points to collect data, this might alleviate the common method variance bias concern to a certain extent. All the items were filled in by individuals; there may still be common method deviations. Therefore, this study uses Harman’s single factor analysis to perform factor analysis on all items. The results show that the proportion of the first principal component factor is 22.791%, which is far less than the 50% criterion. At the same time, the confirmatory factor analysis results in Table 2 show that the single-factor model fits poorly, which further indicates that the influence of common method variance bias in this study is relatively small.

We also tested the reliability and validity of the construct resented in Table 1. The factor loading of each item was greater than the threshold value of 0.60. Similarly, Cronbach’s alpha, and composite reliability measures for each of the constructs were higher than the recommended value of 0.7. Moreover, the average variance extracted for each construct was higher than recommended value of 0.5.

**TABLE 1:** Reliability and validity of the construct.

Constructs	Loading	Alpha	CR	AVE
				
	0.64			
	0.68			
	0.81			
CM	0.78	0.87	0.89	0.5
	0.71			
	0.69			
	0.60			
	0.75			
	0.69			
	0.8			
CP	0.67	0.82	0.91	0.52
	0.74			
	0.71			
	0.69			
	0.72			
	0.75			
	0.71			
WM	0.72	0.88	0.91	0.53
	0.76			
	0.69			
	0.75			
	0.69			
	0.73			
	0.69			
	0.79			
SS	0.87	0.90	0.92	0.67
	0.84			
	0.86			
	0.83			
	0.79			
PR	0.74	0.80	0.88	0.61
	0.8			
	0.77			
	0.79			

*CM, negative customer treatment; CP, positive customer treatment; WM, working meaningfulness; PR, psychological resilience; SS, service sabotage.*

Using confirmatory factor analysis to test the discriminative validity of the questionnaire, as can be seen in Table 2, the five-factor model provided a good fit to the data (χ^2^/df = 1.574, IFI = 0.906, TLI = 0.897, CFI = 0.905, RMSEA = 0.043) compared with other models. It showed that the questionnaire in this study had a good discriminative validity. Hence, the scale fulfills the reliability and validity requirements.

**TABLE 2:** Model of the confirmatory factor analysis.

Model	*χ^2^/df*	*IFI*	*TLI*	*CFI*	*RMSEA*
Five-factor model	1.57	0.91	0.90	0.91	0.05
Four-factor model	2.61	0.74	0.71	0.73	0.08
Three-factor model	2.68	0.72	0.70	0.72	0.08
Two-factor model	3.35	0.61	0.58	0.61	0.10
One-factor model	3.89	0.52	0.48	0.51	0.11

### Description Statistics

The mean, standard deviation, and correlation coefficient of each variable are shown in Table 3. Negative customer treatment and service sabotage were positively correlated (*r* = 0.47, *p* < 0.001); negative customer treatment and work meaningfulness were negatively correlated (*r* = −0.135, *p* < 0.001); work meaningfulness and service sabotage were negatively correlated (*r* = −0.25, *p* < 0.001), indicating that some hypotheses from this study had been initially supported. At the same time, the correlation coefficients among the variables in this study are all less than 0.7, and the VIF values of the multicollinearity test on the regression model are all less than 10, indicating that there is no multicollinearity problem in this study.

**TABLE 3:** Descriptive statistics and correlations.

Variables	Mean	*SD*	1	2	3	4	5	6	7	8	9	10
1. Sex	1.36	0.48	1.00									
2. Age	3.30	0.63	0.00	1.00								
3. Edu	2.97	0.99	−0.17%***	−0.19%***	1.00							
4. Marriage	1.69	0.50	–0.05	0.31%***	–0.04	1.00						
5. Tenure	2.92	0.90	–0.09	0.45%***	0.06	0.30%***	1.00					
6. CM	2.61	0.65	–0.04	–0.10	0.10%*	0.00	–0.08	1.00				
7. CP	2.66	0.72	0.12%*	0.01	–0.06	0.02	–0.01	0.07	1.00			
8. WM	3.58	0.63	−0.12%**	0.04	0.04	0.04	0.14%**	−0.14%**	−0.66%***	1.00		
9. PR	3.47	0.68	−0.18%***	0.02	–0.02	–0.07	0.01	–0.03	−0.48%***	0.62%***	1.00	
10. SS	2.13	0.95	0.02	−0.12%**	0.13%**	–0.04	–0.08	0.468%***	0.03	−0.25%***	−0.22%***	1.00

N = 258; ***p < 0.001; **p < 0.01; *p < 0.05. CM, negative customer treatment; CP, positive customer treatment; WM, working meaningfulness; PR, psychological resilience; SS, service sabotage.

### Hypothesis Testing

This study used hierarchical multiple regression to test the relevant hypotheses, and the relevant results are shown in Table 4. From the M2 in Table 3, it can be seen that there is a significant positive correlation between negative customer treatment and service sabotage (β = 0.67, *p* < 0.001). Thus, Hypothesis 1 was supported.

**TABLE 4:** Results of hypothesis testing.

	M1	M2	M3	M4	M5	M6
	SS	SS	WK	SS	SS	SS
SEX	0.06	0.09	−0.16%*	0.04	0.07	–0.04
AGE	–0.12	–0.08	–0.03	–0.08	–0.09	–0.08
EDU	0.12%*	0.08	0.01	0.08	0.08	0.11%*
MAR	0.01	–0.03	0.01	–0.03	–0.01	0.01
JOB	–0.05	–0.02	0.09%*	0.01	–0.02	–0.03
CM		0.67%***	−0.13%**	0.63%***	0.63%***	
WM				−0.28%***		−0.37%***
CP					–0.04	
CM*CP					−0.13%***	
PR						–0.14
WM*PR						−0.15%***
Constant	2.21%***	0.37	3.91%***	1.47%**	0.66	4.10%***
R2	0.03	0.23	0.05	0.27	0.26	0.12

N = 258; ***p < 0.001; **p < 0.01; *p < 0.05. CM, negative customer treatment; CP, positive customer treatment; WM, working meaningfulness; PR, psychological resilience; SS, service sabotage.

The test of the mediation effect follows the recommendation to adopt the stepwise method to test. First, the M2 in Table 4 shows that negative customer treatment was significantly and positively associated with service sabotage (β = 0.67, *p* < 0.001). Secondly, the M3 in Table 3 shows that negative customer treatment is significantly and negatively associated with work meaningfulness (β = −0.13, *p* < 0.01). Finally, M4 in Table 3 shows that negative customer treatment is significantly and positively associated with service sabotage (β = 0.63, *p* < 0.001) when the mediator is added, and the work meaningfulness was significantly and negatively associated with service sabotage (β = −0.28, *p* < 0.001).So, the partial mediation exists if the effect of the independent variable on the dependent variable becomes weak (when the mediator is added) (0.63 < 0.67). Thus, Hypothesis 2 was supported.

**FIGURE 2:**
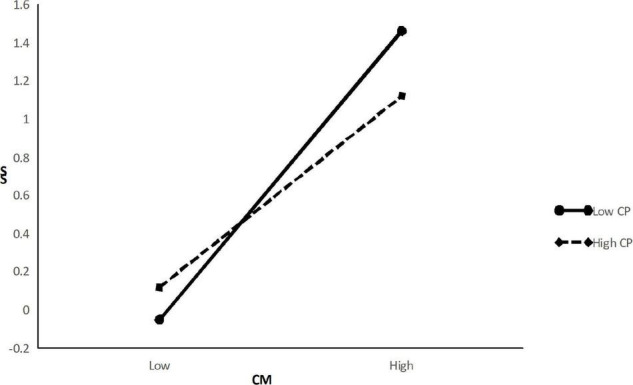
Moderation effects of CP on the CM-SS relationship.

To test Hypothesis 3, we first centered all primary predictor variables before computing the cross-product term. The M5 in Table 4 shows that the interaction term for negative customer treatment and positive customer treatment was significantly associated with service sabotage (β = −0.13, *p* < 0.001). To clarify the moderating effect, this study plotted the interaction by using Aiken and West (1991) method of computing slopes one standard deviation above and below the mean of positive customer treatment. According to Figure 2, it can be seen that negative customer treatment has a weaker positive relationship with service sabotage when positive customer treatment is high than when it is low. Thus, Hypothesis 3 was supported.

**FIGURE 3:**
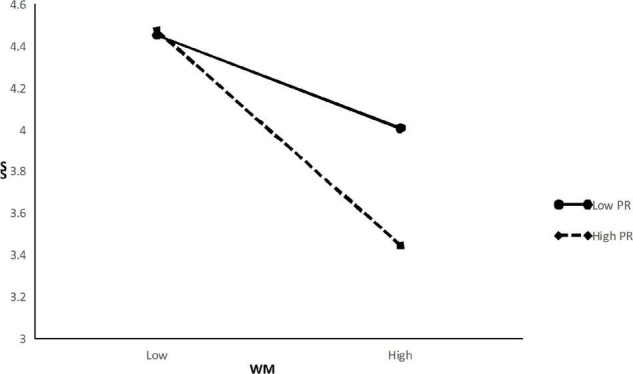
Moderation effects of PR on the WM-SS relationship.

To test Hypothesis 4, this study adopted the same method used in Hypothesis 3. The M6 in Table 4 shows that the interaction term for negative customer treatment and positive customer treatment was significantly associated with service sabotage (β = −0.15, *p* < 0.001). According to Figure 3, it can be seen that work meaningfulness has a weaker positive relationship with service sabotage when psychology resilience is high than when it is low. Thus, Hypothesis 4 was supported.

## Discussion

With the rapid emergence of platform economy, gig workers have become a critical component in the workplace (Fleming, 2017; Guillaume et al., 2019). Although research on the consequences of negative customer treatment has focused on negative customer treatment that occurs to normal employees, less attention has been given to exploring the negative customer treatment experience of gig workers (Baranik et al., 2017; Hu et al., 2018; Cheng et al., 2020). As more and more gig workers choose to work for platform-based service organizations, successful interaction between gig workers and customers is critical for platform success. The primary objectives of this research were to investigate how gig workers respond to negative customer treatment and why this may also occur for gig workers.

Employees’ negative response to customer mistreatment is a robust finding in the organizational behavior research (Chi et al., 2013; Cheng et al., 2020). For example, (Skarlicki et al., 2008) suggested that negative customer treatment is positively related with sabotage from the perspective of moral justice because it violates the principle of justice interaction. Researches on gig workers’ response to negative customer treatment, however, are relatively scarce. For regular employees, they are expected to suppress negative emotions and express a positive attitude and behavior to obey the rules of the organization (Grandey et al., 2004). Otherwise, employees may be subject to be punished for destructive responses to uncivilized customers (Groth and Grandey, 2012). However, gig workers have transformed from being employed into self-employed. They provide services and obtain rewards on the platform, which leads to a simple buying and selling relationship between the platform and gig workers. This new employment model helps to reduce the inequality between gig workers and customers. An important research question concerns the effect of negative customer treatment for gig workers. We expect that gig workers are more sensitive to negative customer treatment, perceived as unfair interpersonal treatment, and more likely to sabotage their services. Our results provided support for our hypothesis, which revealed that when gig workers experience negative customer treatment, they are likely to choose engage in sabotage against customers. This is consistent with previous research on customer mistreatment (Arvan et al., 2020; Lee et al., 2020; Park and Kim, 2020). In the present research, we broaden the research related with the consequence of customer mistreatment by focusing on gig workers.

We also explored the mediating role of work meaningfulness in terms of the association between negative customer treatment and service sabotage. During the process of providing services, the interpersonal interaction between gig workers and customers is one factor that may affect work meaningfulness (Bailey et al., 2017). On the basis of COR theory perspective, gig workers invest their limited resources into interaction with customers to obtain customer recognition and praise of their service (Brotheridge and Grandey, 2002). Customers’ mistreatment behavior, however, allows gig workers unable to get the benefit from the resources they invested; they will find their own work did not bring value to others and themselves, thus resulting in a decline in perceived work meaningfulness. Given that work meaningfulness is one of the most critical psychological factors that influence individuals’ behavior (Humphrey et al., 2007). We find that when gig workers face low levels of work meaningfulness caused by negative customer treatment, they prone to take on sabotage against customer. It also demonstrated that work meaningfulness acts as a mediation role between the association of negative customer treatment and sabotage against customers. Pervious negative customer treatment literature focus on the emotional mechanism to explain its impact on individuals’ behavior and attitudes, few researches had examined the mediating mechanism of work meaningfulness. Our research and thus broadens prior research on the effect of negative customer treatment.

In addition, our research also explored which factor could mitigate the effect of negative customer treatment on reduced work meaningfulness and sabotage against customers. We argue that positive customer treatment can counterbalance the effect of negative customer treatment on sabotage against customers because positive customer treatment may increase individual core resources and counteract the pressure generated by negative customer treatment. In terms of mitigating the effect of work meaningfulness on sabotage against customer, we find that trait of psychological resilience can moderate the association between work meaningfulness and sabotage against customer. Psychological resilience could benefit individuals through better withstanding pressure and recover from adversity, which can be also a core resource (Cooper et al., 2014). The result demonstrated that positive customer treatment moderates the impact of negative customer treatment on service sabotage. Trait resilience can also moderate the effect of low-level work meaningfulness resulting from negative customer treatment on service sabotage.

### Theoretical Contribution

Our research makes several theoretical contributions. First, our research addressed and confirmed an association between negative customer treatment and service sabotage among gig workers, which deepens the negative impact of customer mistreatment. Previous negative customer treatment literature focused on regular employees, and to our knowledge, no studies explored how gig worker response to customer mistreatment (Booth et al., 2018; Amarnani et al., 2019; Cheng et al., 2020; Lavelle et al., 2021). In the context of the platform economy, the relationships between organizations and gig workers are characterized by poor employment relationships, which is cooperative-oriented rather than traditional strong control relationships (Fleming, 2017). Besides, the status difference between employees and customers has been reduced largely (Ashford et al., 2018). Therefore, research is further necessary to explore gig workers response to negative customer treatment. Our results demonstrated that the high frequency of gig workers exposed to negative customer treatment, they were found to more likely engage in service sabotage, which enriched previous literature related to negative customer treatment (Baranik et al., 2017; Hu et al., 2018; Cheng et al., 2020).

Second, we evaluated the roles of work meaningfulness as a mediator of the relationship between negative customer treatment and service sabotage based on the perspective of COR theory. For gig workers, they seek to realize flexible and autonomous and hope to realize their self-worth through working hard (Sessions et al., 2021). Prior research on negative customer treatment suggests that cognitive mechanism such as self-esteem and rumination could explain its effect on employees, ignoring the significant mechanism of work meaningfulness (Wang et al., 2013; Amarnani et al., 2019). Our research bridges this research gap by exploring the role of work meaningfulness in explaining gig workers’ response to customer mistreatment. Our results demonstrated that work meaningfulness mediated the association between negative customer treatment and their sabotage against customer, which broadens the academic understanding of the mechanism of negative customer treatment and enrich the research context of COR theory. This provides a new theoretical perspective and opening the “black box” of the influence of negative customer treatment on gig workers service sabotage.

Finally, our research also explored the boundary condition with respect to the relationship between negative customer treatment and service sabotage, which can be used to address the research question regarding how to alleviate the effect of negative customer treatment on individual service performance. On the one hand, our results suggest that positive customer treatment can weaken the effect of negative customer treatment on service sabotage, which breaks down previous studies that focused only on the effects of negative customer treatment on employees (Sommovigo et al., 2020). Based on the diversity of employee customer interactions, our research further explores the role of positive customer treatment to alleviate the impact of negative customer treatment on individual service performance. It also contributes to theoretically understanding the buffering effect of negative customer treatment on individual service performance. On the other hand, by introducing the personal trait of psychological resilience, our research suggested that employees with higher levels of psychological resilience are more able to effectively deal with the negative impact of customer treatment on work meaningfulness and their service sabotage. Our research expanded the current studies on customer treatment and responded to the call of Harris and Ogbonna (2006) and Chi et al. (2018) to explore moderation to prevent decreased service performance.

### Practical Implications

The present research provides important insights for platform managers who seek to improve service quality and obtain customers’ loyalty. However, employees sabotage behavior directed at customers not only damages customer satisfaction, but also affects the reputation of the platform. Our results suggested that negative customer treatment can be a major concern that threatening service performance because it induced a decreased gig workers’ work meaningfulness perception. One possible strategy for platform managers to prevent gig workers from engaging in service sabotage is to avoid employees experiencing high frequency of customer mistreatment. Organization managers could provide the training opportunity to employees because some mistreated behavior is caused by poor service. Enhanced service capability allows employees to cope with customer demands more qualified. When customers are satisfied with the service, they are less likely to blame or mistreat employees.

Second, managers could monitor employees state and enhance their positive emotion through offering constructive support or advice proactively to them. When employee suffers customer mistreatment, they may question the meaning of the work and fall into negative emotion (Wang et al., 2013). At this time, managers could provide assistance to victims of mistreated such as calm them down, analyses the causes of such negative experience and give some specific suggestions in terms of how response to customer requirements. Through providing support to victims of mistreated, employees could perceive more powerful and energy, which can motivate them to overcome difficulties and provide high quality service.

Another implication for platform managers benefit afforded by psychological resilience traits. Our finding suggests that high psychological resilience employees can inhibit individual tendency to engage in service sabotage when they perceive low levels of work meaningfulness related to negative customer treatment. Notably, high trait psychological resilience can create positive motivational states and behaviors (Mitchell et al., 2019), which allows employees to have more resources to cope with negative customer treatment. Therefore, platform organizations could use personality measures to screen high psychological resilience gig workers, which is valuable in improving platform reputation and service quality.

### Potential Limitations and Future Research Direction

The shortcomings of this study are as follows: First, although the study adopted three stages to collect data, there may still be the influence of the same source deviation because our study relied on self-report measures of all variables. Future research can adopt multi-source and multi-rated data collection, which may provide a more objective method to explore the response of gig workers to negative customer treatment. Second, due to the availability of data collection and the current stage of development of China’s platform-based service industry, the object of this research is mainly online travel and ordering platforms, which has certain limitations. Future research can be expanded with a wider range of research objects, such as some gig workers whose occupations are doctors or consultants located in other countries. Third, although this study explains the impact of negative customer treatment on service sabotage by taking work meaningfulness into consideration, we did not compare the different responses of regular employees and gig workers to customer mistreatment. Future research could seek to collect both regular employee and gig workers data, exploring what the difference in terms of the response to negative customer treatment and why does difference exist. In addition, future research courses could also broaden the outcome of negative customer treatment, such as the work engagement and proactive behaviors.

## Conclusion

The conclusion of this article is as follows: first, negative customer treatment will have a harmful effect on service performance of employees in the organization, and it will activate employees to engage in sabotage against customers. Secondly, negative customer treatment leads to employee’s sabotage against customer through the reduction of their work meaningfulness. Negative customer treatment is often consuming resources and informs that customer question employees’ capability in completing task, which makes them less able to gain work meaningfulness. Therefore, employee cannot gain work meaningfulness from negative customer interaction, and thus prone to retaliate customer by taking on sabotage against customer. Finally, positive customer treatment can reduce the effect of negative customer treatment on sabotage against customer. Trait of resilience could alleviate the effect of reduced work meaningfulness caused by negative customer treatment on sabotage against customers.

## Data Availability Statement

The original contributions presented in the study are included in the article/supplementary material, further inquiries can be directed to the corresponding author.

## Ethics Statement

Ethical review and approval was not required for the study on human participants in accordance with the local legislation and institutional requirements. Written informed consent for participation was not required for this study in accordance with the national legislation and the institutional requirements. Written informed consent was obtained from the individual(s) for the publication of any potentially identifiable images or data included in this article.

## Author Contributions

HX was mainly responsible for research design, data collection, and article writing. LW was responsible for writing the articles and perfecting the research design. LH was responsible for data collection, manuscript polishing, and article writing. LS was in charge of the overall research design. All authors contributed to the article and approved the submitted version.

## Conflict of Interest

The authors declare that the research was conducted in the absence of any commercial or financial relationships that could be construed as a potential conflict of interest.

## Publisher’s Note

All claims expressed in this article are solely those of the authors and do not necessarily represent those of their affiliated organizations, or those of the publisher, the editors and the reviewers. Any product that may be evaluated in this article, or claim that may be made by its manufacturer, is not guaranteed or endorsed by the publisher.
